# Bacterial Community Structure of Panchagavya, a Fermented Liquid Bio-manure, as Revealed by High-Throughput Sequencing of 16S rRNA Gene Amplicons

**DOI:** 10.1128/mra.00941-21

**Published:** 2022-02-03

**Authors:** Dineshkumar Nagarajan, Mukhilan Rajarathnam

**Affiliations:** a Trishul Biotech, Chennai, Tamil Nadu, India; University of Southern California

## Abstract

Panchagavya is a fermented liquid bio-manure that is widely used in India. We aimed to determine its bacterial community structure by sequencing 16S rRNA gene amplicons. These sequences will enhance our understanding of the microbial diversity of panchagavya as well as its potential application in sectors other than agriculture.

## ANNOUNCEMENT

Panchagavya is a liquid bio-manure made through fermentation of five ingredients from cows, namely, dung, urine, milk, curd, and clarified butter (1). Although the nutrient profile, fatty acid profile, proximate content, and phytohormone profile of panchagavya have been well documented (2–4), a comprehensive study to understand the bacterial diversity of panchagavya has not yet been carried out. In this study, the bacterial community structure of panchagavya was determined by sequencing 16S rRNA gene amplicons through an Illumina MiSeq approach.

Commercially available panchagavya from three different batches (J1, J2, and J3) prepared using inputs from the Umbalacheri cow breed, which is native to Tamil Nadu, was sourced from Sri Jayam Trust (Mayiladuthurai, India). The samples (5 mL each) were vortex-mixed at 2,000 rpm and filtered using a 100-μm nylon cell strainer filter (Corning, USA). The filtered samples (1 mL each) were centrifuged at 5,000 rpm for 10 min, and the supernatant was discarded. The pellet was used for DNA extraction with a genomic DNA miniprep kit (Chromous Biotech, India). The concentration and purity of the extracted DNA were checked, and the DNA was used for library generation.

Amplification of the 16S rRNA gene V3-V4 hypervariable region was carried out with 25 ng each of DNA from the J1, J2, and J3 samples. The reaction mixture included KAPA HiFi HotStart Ready Mix (Kapa Biosystems, South Africa) and 100 nM final concentrations of primers 341F (5′-CCTACGGGNGGCWGCAG-3′) and 785R (5′-GACTACHVGGGTATCTAATCC-3′) (5). The PCR involved an initial denaturation at 95°C for 5 min followed by 25 cycles of 95°C for 30 s, 55°C for 45 s, and 72°C for 30 s and a final extension at 72°C for 7 min. The amplicons were purified using AMPure XP beads (Beckman Coulter, USA) to remove unused primers. Eight additional cycles of PCR were performed using Illumina barcoded adapters to prepare the sequencing libraries. The libraries were sequenced using a MiSeq system (Illumina, USA). Data quality was checked using FastQC (http://www.bioinformatics.babraham.ac.uk/projects/fastqc) and MultiQC (6) software. The data were checked for base call quality distribution, percentage of bases with scores above Q20 or Q30, GC content, and sequencing adapter contamination. All of the samples passed the quality control (QC) threshold (>95% with scores of Q20). The numbers of 300-bp paired-end reads generated for the J1, J2, and J3 samples were 103,568, 99,970, and 108,342, respectively. The sequence reads were trimmed (20 bp) from the 5′ end to remove the degenerate primers. The trimmed reads were processed to remove adapter sequences and low-quality bases using Trim Galore (https://www.bioinformatics.babraham.ac.uk/projects/trim_galore). The QC-passed reads were imported into the mothur program (7), and subsequent analyses were performed as described by Kozich et al. (8).

The UCHIME algorithm (9) was used to flag contigs with chimeric regions. A known reference for all of the chimeric sequences was used to identify and remove possible chimeric sequences. The filtered contigs were processed and classified into taxonomical outlines based on the Greengenes v.13.8-99 database (10). Sequences were clustered according to their similarity to one another, and operational taxonomic units (OTUs) were defined based on a similarity threshold (97% similarity) using the mothur phylotype clustering method. After the classification, OTU abundance was estimated (Table 1).

**TABLE 1:** Relative abundance of OTUs in panchagavya samples

Kingdom and phylum	Relative abundance (%) of OTUs in sample:		
	J1	J2	J3
*Bacteria*			
* Bacteroidetes*	46.64	46.98	41.82
* Firmicutes*	23.66	29.11	33.74
* Proteobacteria*	23.27	16.69	15.81
* Actinobacteria*	3.33	3.5	5.18
* Spirochaetes*	1.74	1.91	1.76
* Cyanobacteria*	0.45	0.62	0.54
* Verrucomicrobia*	0.22	0.31	0.32
* Lentisphaerae*	0.16	0.26	0.17
WWE1	0.1	0.18	0.21
*Tenericutes*	0.21	0.13	0.1
TM7	0.1	0.19	0.14
*Archaea*			
* Euryarchaeota*	0.06	0.04	0.13
*Bacteria*			
* Planctomycetes*	0.02	0.02	0.03
* Synergistetes*	0.01	0.02	0.02
* Elusimicrobia*	0.01	0.01	0
* Fusobacteria*	0.01	0.01	0
* Chloroflexi*	0.01	0	0.01

### Data availability.

The high-throughput 16S rRNA gene sequencing data generated in this study were deposited in the European Nucleotide Archive under BioProject accession number PRJEB43256, with GenBank accession numbers ERX5138760, ERX5138761, and ERX5138762.
